# Repeatability and Reproducibility of Anterior Segment Measurements in Normal Eyes Using Dual Scheimpflug Analyzer

**DOI:** 10.4274/tjo.16768

**Published:** 2015-12-05

**Authors:** Zeynep Altıparmak, Ramazan Yağcı, Emre Güler, Zeynel Arslanyılmaz, Metin Canbal, İbrahim F. Hepşen

**Affiliations:** 1 Ulucanlar Eye Training and Research Hospital, Ankara, Turkey; 2 Pamukkale University Faculty of Medicine, Department of Ophthalmology, Denizli, Turkey; 3 Erciş State Hospital, Clinic of Ophthalmology, Van, Turkey; 4 Adıyaman University Training and Research Hospital, Clinic of Ophthalmology, Adıyaman, Turkey; 5 Turgut Özal University Faculty of Medicine, Department of Public Health, Ankara, Turkey; 6 Gazi University Faculty of Medicine, Department of Ophthalmology, Ankara, Turkey

**Keywords:** Galilei, intraobserver, interobserver repeatability, wavefront aberrations

## Abstract

**Objectives:**

To assess the repeatability and reproducibility of anterior segment measurements including aberrometric measurements provided by a dual Scheimpflug analyzer (Galilei) system in normal eyes.

**Materials and Methods:**

Three repeated consecutive measurements were taken by two independent examiners. The following were evaluated: total corneal power and posterior corneal power, corneal higher-order wavefront aberrations (6.0 mm pupil), pachymetry at the central, paracentral, and peripheral zones, and anterior chamber depth (ACD). Repeatability was assessed by calculating the within-subject standard deviation, precision, repeatability, and intraclass correlation coefficient (ICC). Bland-Altman analysis was used for assessing reproducibility.

**Results:**

Thirty eyes of 30 patients were included. The best ICC values were for corneal pachymetry and ACD. For both observers, acceptable ICC was also achieved for the other parameters, the only exceptions being posterior corneal astigmatism and total high order aberration. The 95% LoA (Limits of Agreement) values for all measurements showed small variability between the two examiners.

**Conclusion:**

The Galilei system provided reliable measurements of anterior segment parameters. Therefore, the instrument can be confidently used for routine clinical use and research purposes.

## INTRODUCTION

Anterior segment imaging has significantly improved since the introduction of Scheimpflug cameras into clinical practice. The first rotating Scheimpflug camera, the Pentacam (Oculus, Wetzlar, Germany), became commercially available in 2002 and provided reliable measurements.^1,2,3^

In recent years, a new Scheimpflug-based device, the Galilei dual Scheimpflug analyzer (Ziemer Group, Switzerland), was introduced to the market.^4^ In contrast to the Pentacam, the Galilei includes two rotating Scheimpflug cameras in combination with a Placido topography system. The Galilei uses the Placido disc to provide more accurate anterior curvature topographic data in addition to the data obtained from Scheimpflug cameras.^5^ In addition, the dual camera system derives images from both sides, which minimizes the effect of decentration due to eye movements on corneal pachymetry and posterior corneal curvature measurements.^6^

Low intraobserver and interobserver variability is essential to validate a new instrument before clinical application.^7,8^ In this study we aimed to evaluate the repeatability and reproducibility of the Galilei for anterior segment measurements, including wavefront aberration analysis.

## MATERIALS AND METHODS

This prospective study was performed in accordance with the ethical standards stated in the Declaration of Helsinki and was approved by the local ethics committee. All patients provided informed consent.

The subjects were recruited from the Ophthalmology Department of Turgut Özal University in Ankara, Turkey between October and December 2013. Subjects without previous ocular surgery or trauma, corneal or other ocular diseases, and contact lens use were enrolled in the study. All subjects included in this study had best-corrected visual acuity (BCVA) of -0.10 to 0.10 logMAR, refractive error (in spherical equivalent) within ±1.00 diopters (D) and astigmatism not exceeding 0.50 D.

One eye of each patient was randomly selected. All eyes underwent a comprehensive ophthalmologic examination including corneal topographic analysis with the Galilei dual Scheimpflug analyzer system. Three consecutive measurements were taken by two independent, experienced examiners to assess repeatability and reproducibility. The first examiner was assigned randomly for each case.

All eyes underwent corneal topographic analysis with the Galilei dual Scheimpflug analyzer system (software version 5.2.1), a noninvasive system for measuring and characterizing the anterior segment. The scanning process acquires a series of Scheimpflug images (meridians) and two Placido top-view images, each 90 degrees apart. The anterior cornea, posterior cornea, anterior lens, and iris edges are determined from the Scheimpflug images; the ring edges are detected in the Placido images. One set of height data is derived directly from the Scheimpflug edges, while slope data from the Placido images are transformed into another set of height data. The combined height data are merged and used to create a surface fit of the anterior cornea by means of a proprietary method. All other measurements (posterior cornea, anterior lens, and iris) are derived solely from Scheimpflug data.

All measurements were taken between 10 am and 3 pm with non-dilated pupils in identical lighting conditions. Measurements were performed according to the manufacturer’s guidelines. The device was brought into focus, and the patient’s eye was aligned along the visual axis using a central fixation light. Patients were instructed to blink completely just before each measurement and the device was realigned before each measurement. Patients were randomly assigned to have three consecutive measurements of ocular components by two examiners.

The following measurements were evaluated in this study:

### 1- Mean simulated keratometry (SimK) average:

This is the arithmetic mean of the pair of meridians 90 degrees apart with the greatest difference in average power, at a distance of 0.5 to 2.0 mm from the center. 

### 2- Mean total corneal power (TCP) and total corneal astigmatism (TCA):

Masurements of the power of the anterior and posterior corneal surfaces are obtained through ray tracing rather than the Gaussian optics formula. For each point on the map, the angle of incidence is calculated relative to the anterior surface normal for incoming parallel rays. The angle of refraction is calculated using Snell’s law with refractive index (Z)=1.0 for air and Z=1.376 for the cornea. This angle of refraction is used to determine the nonparallel direction of incoming rays relative to the posterior surface normal and is used to calculate the angle of incidence for the posterior surface. A new angle of refraction is calculated for the posterior surface using Snell’s law with corneal Z=1.376 and aqueous Z=1.336. This final angle of refraction is used to calculate the intersection of the ray along the (0.0) axis and the resultant focal length that is used to determine total power for that point on the map.

The TCA value is the difference between SimK in the steepest meridian (SimKs) and SimK in the flattest meridian (SimKf).

### 3- The mean posterior corneal power (PCP) and posterior corneal astigmatism (PCA):

PCP (derived from the posterior axial curvature map) is the arithmetic mean of the pair of meridians 90 degrees apart with the greatest difference in average power, at a distance of 0.5 to 2.0 mm from the center. The power of the steep and flat meridian is calculated using the cornea (1.376) and aqueous humor (1.336) refractive indexes. The PCA value is the difference between SimKs and SimKf.

### 4- Corneal thickness (CT):

The mean CT in the central zone (0.0 to 4.0 mm), paracentral zone (4.0 to 7.0 mm), and peripheral zone (7.0 to 11.0 mm) was evaluated.

### 5- Anterior chamber depth (ACD):

The ACD is the distance between the corneal endothelium and the anterior surface of the crystalline lens measured perpendicularly to the lens surface. In this study, the ACD value was the mean of all measured Scheimpflug scans.

### 6- Total corneal wavefront aberrations, total corneal higher-order wavefront aberrations, and spherical aberration (SA):

The dual Scheimpflug system displays the total corneal wavefront aberrations calculated from the front and back surfaces, centered on the pupil. The following values were recorded with a 6.0 mm pupil: Total corneal root-mean-square (RMS total), high-order RMS (HO RMS) for the 3rd to 6th orders, and spherical aberration. SA was evaluated in particular because of its importance in implanting aspheric intraocular lenses.

### Statistical Analysis

Statistical analysis was performed using the SPSS for Windows software version 11.5 (SPSS Inc., Chicago, IL, United States). Normality of all data distributions was confirmed by the Kolmogorov-Smirnov test. Then, parametric statistics were applied. The paired t-test was used to analyze the comparison between examiners for each clinical parameter. All tests were two-tailed; level of significance was accepted as α=0.05.

Repeatability for each clinical parameter was assessed using the following statistical parameters: the within-subject standard deviation (Sw) of the three consecutive measurements, precision, repeatability and the intraclass correlation coefficient (ICC). The within-subject standard deviation is a simple way of estimating the size of the measurement error. The precision was defined as (±1.96xSw)^9^ and this parameter indicates how large the range of error of the repeated measurements for 95% of observations is. The repeatability was computed as (2.77xSw); this is another useful way of presenting the range of measurement error.^10^ The ICC is an analysis-of-variance type correlation that measures the relative homogeneity within groups (between the repeated measurements) in ratio to the total variation.^11^ The maximum ICC value is 1.00, and ICC values closer to 1.00 indicate greater reliability. In general, Portney and Watkins12 suggested that ICC values above 0.75 indicate good reliability, but for most clinical measurements ICC should be over 0.90 to ensure reasonable validity. If the value falls below 0.00, the ICC is not valid. Reproducibility was evaluated by Bland-Altman analysis. This method uses graphing to assess whether there is agreement between two observers. The limits of agreement were calculated as the mean difference in measurements obtained by each observer ±1.96xSD of the differences.^9^ This standard deviation is by definition the interobserver range of agreement (1.96 times), with lower values indicating higher reproducibility. The reproducibility is not acceptable if the range of agreement is clinically relevant (error with significant implication in the clinical practice), indicating that the evaluated clinical methodology does not provide repeatable measurements.

Sample-size calculations were performed to check whether the number of subjects included in the study was sufficient to detect a statistical difference among repeated intrasubject measurements. The number of patients included in the study was chosen according to the results from sample-size calculations based on intraobserver ICC. A sample size of 26 subjects with 3 observations per subject achieves 81% power to detect an intraclass correlation of 0.8 under the alternative hypothesis when the intraclass correlation under the null hypothesis is 0.6 using an F-test with a significance level of 0.05.

## RESULTS

Thirty eyes of 30 volunteers (15 male, 15 female) were evaluated in this prospective study. The mean age of the participants was 30.15 ± 5.02 (20-40) years.

### Repeatability

Table 1 shows the repeatability results of corneal power measurements for both examiners. High ICC results were achieved for corneal power measurements of SimK average, PCP, and TCP. However, measurements related to PCA showed less repeatability, with ICC values of 0.602 and 0.576 for examiners 1 and 2, respectively (Table 1). In addition, the measurements of TCA for examiner 1 had a lower ICC value (ICC: 0.680).

Table 2 summaries the repeatability results for CT and ACD measurements. For both examiners, the best ICC values (greater than 0.90) were obtained for the CT and ACD measurements. Central CT (0 to 4 mm) measurement showed the best ICC results (ICC over 0.99).

Table 3 shows the repeatability results for corneal aberrations. All measurements achieved good ICC values (over 0.75), whereas total HO RMS measurements had less repeatability (ICC for examiners 1 and 2 were 0.717 and 0.641, respectively).

### Reproducibility

Table 4 summarizes reproducibility results for the ocular components analyzed by both observers. Overall, the 95% LoA for measured parameters showed that the examiners demonstrated very good agreement with each other. The smallest range of agreement was shown in ACD (0.0594 mm), whereas the largest was for the measurements related to corneal thickness, which were smaller in the central cornea compared to peripheral cornea. The 95% LoA for all measurements showed low variability between measurements of both examiners.

## DISCUSSION

Reliability studies of diagnostic devices are necessary to ensure that the error involved in measurement is small enough to detect actual changes in what is being measured.^13^ The present study was designed to evaluate the repeatability and reproducibility of the anterior segment measurements provided by the Galilei dual Scheimpflug+Placido corneal topographer in healthy corneas.

Menassa et al.^5^ and Wang et al.^14^ showed a high ICC (over 0.99) for the repeatability of corneal pachymetric measurements using the Galilei Scheimpflug system in normal corneas. Savini et al.^15^ reported excellent repeatability for measurements of central and thinnest CT in both normal and postrefractive corneas (ICC was over 0.99 for both groups). In the current study, we found similar ICC values (greater than 0.99) for the repeatability of corneal pachymetric in normal corneas.

Menassa et al.^5^ reported that central corneal pachymetry readings with the Galilei and a scanning-slit topographer (Orbscan II, Bausch&Lomb) showed high reproducibility in normal corneas. In the present study, we found acceptable reproducibility for pachymetry readings for normal corneas.

Shankar et al.^1^ reported good repeatability for central corneal thickness, whereas the peripheral pachymetry repeatability was poor using the single Scheimpflug camera system. In contrast, Wang et al.^14^ showed excellent repeatability for both central and peripheral corneal pachymetry with the dual Scheimpflug system. In this study, we showed excellent repeatability for central and peripheral CT measurements. This may be associated with the dual-channel Scheimpflug cameras implemented by the system. If the alignment varies between measurements, different thicknesses will be detected depending on the camera location and magnitude of decentration. In contrast, measurement values obtained by averaging data from two cameras should minimize the problems caused by altered corneal position because such a shift will produce a thinner measurement by one camera and a correspondingly thicker measurement by the other camera (J.R. Lewis, MD, et al., “Comparison of Response to Misalignment in Pachymetry Measurement Between Single- and Dual-Scheimpflug Devices” presented at the ASCRS Symposium on Cataract, IOL and Refractive Surgery, San Francisco, California, USA, April 2009).

In this study, corneal power measurements including SimK average, PCP, and TCP yielded high repeatability similar to those previously reported by Wang et al.^14^ and Savini et al.^15^ Aramberri et al.^16^ found comparable repeatability for posterior corneal astigmatism between Galilei and Pentacam devices, with ICC values of 0.725 and 0.776, respectively. Szalai et al.^17^ evaluated the Pentacam HR device but had lower astigmatism repeatability (Sw: 0.066). According to our results, the repeatability of PCA was slightly lower (ICC values less than 0.602) compared to the Aramberri et al.^16^ study and was considerably lower compared to Wang et al.,^14^ whose ICC value was 0.913.

When considering the reproducibility of corneal power measurements, the Galilei achieved high ICC values, similar to those previously reported by Wang et al.^14^ The reproducibility results were also comparable to the Pentacam reported by Aramberri et al.^16^

ACD measurement has become necessary in cataract and refractive surgery for sophisticated IOL power calculation methods and phakic IOL implantation. Previous studies reported excellent repeatability for ACD measurements using the Galilei (ICC over 0.99).^14,15^ The current study confirms the excellent repeatability of ACD measurements, achieving ICC values over 0.99. In addition, the Galilei yielded high reproducibility for ACD measurements, similar to that reported by Fukuda et al.^18^

Wang et al.^14^ reported high repeatability for SA (ICC: 0.981) and total HO RMS (ICC: 0.858). Savini et al.^15^ reported excellent repeatability for measurements of SA in normal and postrefractive corneas (ICC was consistently over 0.941). In this study we showed good ICC values (over 0.75) for all measured wavefront aberrations, whereas total HO RMS measurements had less repeatability (ICC less than 0.717). In addition, we found acceptable reproducibility for the measurements related to wavefront aberrations.

Although we did not primarily aim to compare the repeatability of the Galilei with other Scheimplug-based anterior segment imaging devices, a literature review indicates similar (and sometimes better) repeatability for the Galilei analyzer. Our data also confirm the results in previous studies of the repeatability and reproducibility of Galilei measurements. Therefore, the Galilei dual Scheimpflug analyzer can be confidently used for routine clinical use and research purposes.

## Figures and Tables

**Table 1:**
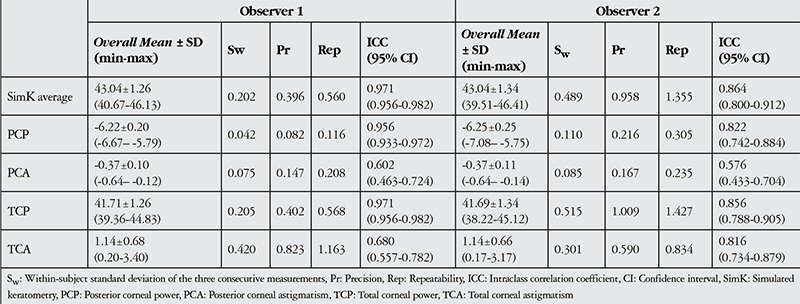
Repeatability for corneal power measurements

**Table 2:**
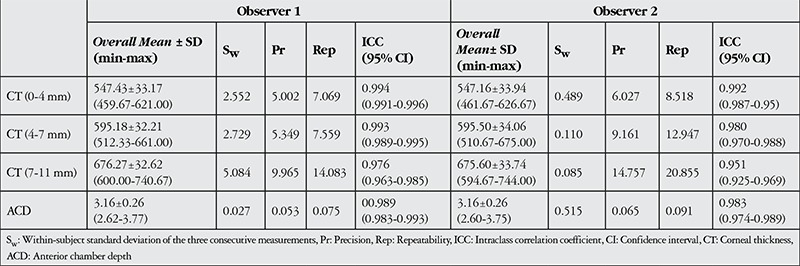
Summary of repeatability results for corneal thickness and anterior chamber depth measurements

**Table 3:**
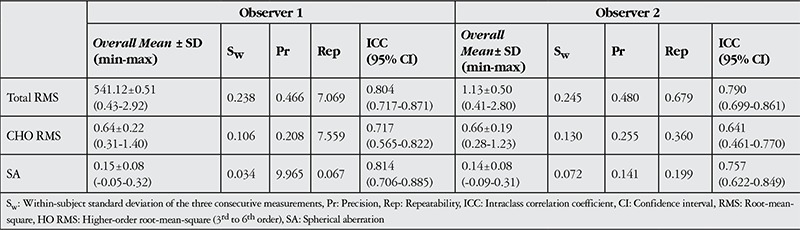
Summary of repeatability results for corneal wavefront measurements

**Table 4:**
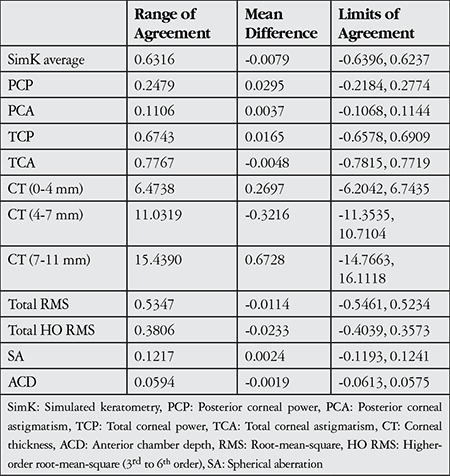
Summary of reproducibility results for the clinical parameters
